# Parity and Risk of Death From Lung Cancer Among a Cohort of Premenopausal Parous Women in Taiwan

**DOI:** 10.2188/jea.JE20110123

**Published:** 2012-07-05

**Authors:** Meng-Hsuan Cheng, Shang-Shyue Tsai, Chih-Cheng Chen, Shu-Chen Ho, Hui-Fen Chiu, Trong-Neng Wu, Chun-Yuh Yang

**Affiliations:** 1 Division of Pulmonary and Critical Medicine, Department of Internal Medicine, Kaohsiung Medical University Hospital, Kaohsiung, Taiwan; 2 Graduate Institute of Medicine, Kaohsiung Medical University, Kaohsiung, Taiwan; 3 Department of Internal Medicine, Ping-Tung Hospital, Department of Health, Ping-Tung, Taiwan; 4 Department of Healthcare Administration, I-Shou University, Kaohsiung, Taiwan; 5 Department of Pediatrics, Kaohsiung Chang-Gung Memorial Hospital and Chang-Gung University, College of Medicine, Kaohsiung, Taiwan; 6 Department of Public Health, College of Health Sciences, Kaohsiung Medical University, Kaohsiung, Taiwan; 7 Institute of Pharmacology, College of Medicine, Kaohsiung Medical University, Kaohsiung, Taiwan; 8 Department of Public Health, China Medical University, Taichung, Taiwan; 9 Division of Environmental Health and Occupational Medicine, National Health Research Institute, Miaoli, Taiwan

**Keywords:** lung cancer, parity, mortality, cohort study

## Abstract

**Background:**

We examined the association between parity and risk of lung cancer.

**Methods:**

The study cohort consisted of all women with a record of a first singleton birth in the Taiwanese Birth Register between 1978 and 1987. We tracked each woman from the time of their first childbirth to 31 December 2009. Follow-up was terminated when the mother died, when she reached age 50 years, or on 31 December 2009, whichever occurred first. The vital status of mothers was ascertained by linking records with the computerized mortality database. Cox proportional hazard regression models were used to estimate hazard ratios (HRs) for death from lung cancer associated with parity.

**Results:**

There were 1375 lung cancer deaths during 32 243 637.08 person-years of follow-up. The mortality rate of lung cancer was 4.26 cases per 100 000 person-years. As compared with women who had given birth to only 1 child, the adjusted HR was 1.13 (95% CI, 0.94–1.35) for women who had 2 children, 1.10 (0.91–1.33) for those who had 3 children, and 1.22 (0.96–1.54) for those who had 4 or more children.

**Conclusions:**

The findings suggest that premenopausal women of higher parity tended to have an increased risk of lung cancer, although the trend was not statistically significant.

## INTRODUCTION

In Taiwan, lung cancer is the second leading cause of cancer mortality for men and the leading cause for women. In 2010, the age-adjusted mortality rate for lung cancer was 35.10 per 100 000 men and 17.1 per 100 000 women.^1^

Smoking is a strong causal risk factor for female lung cancer.^2^ However, because female lung cancer rates are relatively high, and female smoking prevalence is low, in Taiwan, smoking cannot fully explain the epidemiologic characteristic of female lung cancer in that country.^3^ In Taiwan, the proportion of female lung cancer patients who were smokers was only 7.14%,^3^ the prevalence of smoking among women was only about 4.2%,^4^ and a significant proportion of lung cancer cases occurred among nonsmokers. Because of the very low smoking prevalence among women, we believe that the Taiwanese female population (ie, the primarily nonsmoking population) is an appropriate group for studies of the effect of other types of exposures (including reproductive factors) on lung cancer risk. We have previously studied the female population to assess the relationship between air pollution and lung cancer.^5^^,^^6^

Although smoking is a strong risk factor for lung cancer, the etiology of lung cancer among nonsmokers is less well understood.^7^ Evidence that lung cancer among nonsmokers is a distinct clinicopathologic entity has been reviewed elsewhere.^8^ Among individuals with lung cancer, there are higher proportions of nonsmokers and adenocarcinomas in women than in men.^7^ In addition, some studies suggest that women are more susceptible than men to tobacco carcinogens.^9^^–^^11^ These findings indicate that sex-specific hormones may have a role in lung cancer pathogenesis in women.^12^ The influence of sex hormones on lung cancer risk is supported by the existence of estrogen receptors in both normal and neoplastic human lung tissues.^13^^,^^14^

Serum estrogen levels have been reported to rise about 100-fold during pregnancy.^15^ Higher parity is therefore associated with increased lifetime exposure to estrogen. Only a few studies examined the role of parity in the development of lung cancer, and the results have been mixed. Some studies found inverse associations between parity and lung cancer risk,^16^^–^^22^ while other epidemiologic studies reported null^23^^–^^25^ or positive associations.^26^

Two of the aforementioned studies of the relationship between parity and lung cancer risk were limited to postmenopausal women.^16^^,^^23^ Other studies did not divide the study sample into pre- and postmenopausal women because the number of premenopausal lung cancer cases was insufficient to achieve adequate statistical power. Thus, these studies included both pre- and postmenopausal women in the sample, and primarily the latter.^17^^–^^22^^,^^24^^–^^26^ Only 1 study examined the relationship between parity and lung cancer in pre- and postmenopausal women separately^27^: Schwartz et al reported that increasing parity was associated with a modest increase in lung cancer risk among pre/perimenopausal women but not in postmenopausal women.

Although the results have been inconsistent, previous epidemiologic studies indicate that selected reproductive factors, particularly parity, might reduce lung cancer risk in women. The incidence of lung cancer in premenopausal women is low; therefore, the effect of parity on the development of lung cancer in this group is easily lost in overall analyses.

In our previous studies, we found a significant association between higher parity and protection against ovarian,^28^ pancreatic,^29^ and liver cancers.^30^ The objective of the present study was to explore further the effect of parity on the risk of lung cancer in a young cohort of 1 292 462 premenopausal parous Taiwanese women who were followed over a period of 32 years. This is one in a series of studies that use the same cohort to investigate the relationship between parity and risk of cancer at various sites.

## METHODS

### Data source

All births must be reported to the Taiwan Local Household Birth Registry, which is managed by the Ministry of Interior (MOI), within 15 days of delivery. The MOI has released a computerized Birth Registration Database since 1978. Birth-related information obtained includes birth date, single/multiple pregnancy, gestational age, infant sex, birth weight, and parental information, including maternal age, marital status, educational level, and maternal parity. The birth registration data are considered complete, reliable, and accurate because most deliveries in Taiwan take place in either a hospital or clinic,^31^ birth certificates are completed by physicians attending the delivery, and all live births must be registered at local household registration offices.^28^^–^^30^

### Study population

The data in this analysis were derived from a population-based prospective study of 1 292 462 women with a record of a first singleton birth between 1 January 1978 and 31 December 1987 in the Birth Registration Database. A detailed description of this cohort has been published elsewhere.^28^^–^^30^ Using personal identification numbers, data on any subsequent births were linked to the Birth Registration Database.

### Follow-up

Using personal identification numbers, we followed the mothers via linkage with the computerized Taiwan Mortality Database to identify dates and causes of death. Follow-up continued until date of death, age 50 years (ie, postmenopausal women),^32^ or 31 December 2009, whichever came first. Because it is mandatory to register death certificates at local household registration offices, the mortality statistics in Taiwan are considered to be highly accurate and complete.^28^^–^^30^

### Statistical analysis

We categorized parity (the number of children recorded in the last childbirth record of each woman registered during follow-up) into 4 categories: 1, 2, 3, and 4 or more. We compared selected baseline characteristics of the cohort with regard to parity using chi-square tests and analysis of variance, as appropriate. For each member of the cohort, person-years of follow-up were computed from the date of a woman's first childbirth to date of death, age 50 years,^32^ or 31 December 2009, whichever occurred first. Death rates were calculated by dividing number of lung cancer deaths (ICD-9 code 162) by number of person-years of follow-up. Cox proportional hazards regression models were used to estimate hazard ratios (HRs) for lung cancer death associated with parity. The 95% CIs for HRs were also calculated. We used 2 Cox proportional hazards models: an age-adjusted model and a multivariate-adjusted model that was additionally adjusted for marital status (married, unmarried), years of schooling (≤9, >9 years), and birthplace (hospital/clinic, home/other). The proportional hazards assumption was assessed for all the above-mentioned variables, and no violations were observed. To test for trends in risk with increasing levels of the exposures of interest, we assigned the categorical variables their ordinal number for parity and the category median for age at first birth and then fitted the assigned values for each risk factor as a continuous variable in the risk models. We then evaluated the statistical significance of the corresponding coefficient using the Wald test.^33^ All calculations were performed using the SAS statistical package (version 8.02, SAS Institute Inc., Cary, NC, USA), and all *P* values quoted are 2-sided. A *P* value less than 0.05 was considered statistically significant.

## RESULTS

Complete information from 1 292 462 primiparous women was included in the analysis. A total of 32 243 637.08 person-years were observed during the follow-up period from the time of first childbirth to 31 December 2009. The mean follow-up period was 25.5 (SD, 3.24) years. There were 1375 lung cancer deaths, yielding a mortality rate of 4.26 cases per 100 000 person-years.

Table 1 shows the baseline characteristics of the study population by parity. Compared with women who had given birth to only 1 child, women with 4 or more children were more likely to have a lower educational level, to be younger at first birth, and to have a lower rate of being born in a hospital or clinic.

**Table 1.** Demographic characteristics of the study cohort

	Parity				*P*-value

	1 (*n* = 157 207)	2 (*n* = 564 727)	3 (*n* = 436 250)	4+ (*n* = 134 278)	
Age at recruitment (first birth)	26.37 ± 4.41	24.86 ± 3.30	23.49 ± 2.95	22.43 ± 2.93	<0.0001
Marital status					
Married	146 022 (92.89)	554 810 (98.24)	429 239 (98.39)	130 544 (97.22)	<0.0001
Not married	11 185 (7.11)	9917 (1.76)	7011 (1.61)	3734 (2.78)	
Years of schooling					
≤9	72 090 (45.86)	258 361 (45.75)	285 737 (65.50)	106 330 (79.19)	<0.0001
>9	85 117 (54.14)	306 366 (54.25)	150 513 (34.50)	27 948 (20.81)	
Birthplace					
Hospital/clinic	153 167 (97.43)	553 930 (98.09)	416 492 (98.47)	122 336 (91.11)	<0.0001
Home/other	4040 (2.57)	10 797 (1.91)	19 758 (4.53)	11 942 (8.89)	

Table 2 shows the HRs for lung cancer death by parity. After adjustment for age at first birth, the HR for lung cancer death was 1.10 (95% CI, 0.92–1.31) for women who had 2 children, 1.09 (0.91–1.32) for women who had 3 children, and 1.23 (95% CI = 0.98–1.55) for women with 4 or more births, as compared with women who had given birth to 1 child. There was a nonsignificant increasing trend in the HRs for lung cancer with increasing parity (*P* for trend = 0.15). In the multivariate-adjusted model (ie, adjusted for age at first birth, marital status, years of schooling, and birth place), the HRs were only slightly altered. The adjusted HR was 1.13 (0.94–1.35) for women who had 2 children, 1.10 (0.91–1.33) for women who had 3 children, and 1.22 (95% CI = 0.96–1.54) for women with 4 or more births, as compared with women who had given birth to 1 child. Increasing parity appeared to be associated with a modestly increased risk of lung cancer. However, these results were not statistically significant, and there was no clear trend with parity (*P* = 0.25).

**Table 2.** Association between parity and relative risk of death from lung cancer over a 32-year follow-up period

Parity	No. of subjects	Follow-up (person-years)	No. of deaths from lung cancer (per 100 000 person-years)	Age-adjusted HR (95% CI)	Multivariate-adjusted HR (95% CI)*
1	157 207	3 626 856.83	152 (4.19)	1.00	1.00
2	564 727	13 847 350.50	605 (4.37)	1.10 (0.92–1.31)	1.13 (0.94–1.35)
3	436 250	11 200 602.33	460 (4.11)	1.09 (0.91–1.32)	1.10 (0.91–1.33)
4+	134 278	3 568 827.42	158 (4.43)	1.23 (0.98–1.55)	1.22 (0.96–1.54)
				*P* = 0.15 for linear trend	*P* = 0.25 for linear trend
Continuous	1 292 462	32 243 637.08	1375 (4.26)	1.05 (0.99–1.12)	1.04 (0.98–1.11)

*Adjusted for age at first birth, marital status, years of education, and birthplace.

HR: hazard ratio.

## DISCUSSION

To our knowledge, this is the largest cohort (*n* = 1 292 462 women) study of the relationship between parity and lung cancer risk. In this prospective cohort study, we found that women with higher parity tended to have an increased risk of lung cancer, but the trend was not statistically significant. Our finding of a possible increase in lung cancer risk associated with higher parity is in agreement with the results of some studies^26^^,^^27^; however, other studies have reported a lower risk^16^^–^^22^ or no association^23^^–^^25^ with higher parity.

Pregnancy elevates serum estrogen levels by about 100 fold.^15^ Increasing parity is associated with an overall increase in lifetime exposure to sex hormones. Although there is experimental evidence that estrogen receptors and other steroid hormone receptors are present in both normal lung tissue and lung tumors,^13^^,^^14^ their role in lung carcinogenesis is unclear. Estrogens may influence lung cancer development, either through direct promotion of cellular proliferation in the lung or as a result of an effect on lung-carcinogen metabolism or the development of lung diseases that predispose to lung cancer.^12^^,^^34^ Estrogens have also been implicated as a cause of lung cancer in the absence of receptor activation. They may represent direct-acting carcinogens, after metabolic activation to catechol estrogens, which can form DNA adducts.^35^ These and related findings have stimulated renewed interest in the possible role of steroid hormones in promoting lung cancer.^36^ Although evidence suggests that greater exposure to endogenous estrogen would, if anything, increase lung cancer risk, in general, this hypothesis has not been strongly supported by results from epidemiologic studies.^17^

In our study, all lung cancer deaths occurred among premenopausal women. Mean age at death due to lung cancer was 43.61 ± 5.11 years among participants in this study, which is younger than in previous studies. Mean duration from last delivery to lung cancer death was approximately 15.13 years (mean age [SD] at birth of last child, 28.48 ± 4.05 years). Hormonal conditions resulting from the events of pregnancy or delivery may have had a role in these premenopausal lung cancer cases. Our finding of a possible positive association between lung cancer risk and parity might explain the link to increased hormonal production after multiple childbirths, although a previous study found that parity was associated with lower levels of reproductive hormones.^37^

Because the incidence of lung cancer among premenopausal women is low, the effect of parity on lung cancer development in this group is easily lost in overall analyses. In the literature, only 1 case-control study investigated the link between parity and lung cancer among pre- and postmenopausal women separately^27^: Schwartz et al reported a modest increase in lung cancer risk among pre- and perimenopausal women. Their study, however, was based on a very small sample of only 44 cases and therefore had limited statistical power. To our knowledge, ours is the first cohort study to show a positive association between increasing parity and risk of lung cancer death among premenopausal women. Nonetheless, because the literature to date lacks consistent evidence of an association between parity and lung cancer mortality risks, we cannot exclude the possibility that this is a chance finding. Further studies are required to increase understanding of the effect of parity on lung cancer risk.

In the event of a death, Taiwanese law requires the decedent's family to obtain a death certificate from the hospital or local community clinic. The certificate must then be submitted to the household registration office, which then removes the decedent from the family register. The death certificate must be completed and certified by a physician in Taiwan and is required for the decedent to be interred or cremated. Because it is mandatory to register all deaths at the local household registration office in the decedent's jurisdiction, Taiwan's death records are regarded as reliable and complete.^28^^–^^30^ Moreover, this study is highly unlikely to be plagued by selection bias, because access to health care is universal in Taiwan, vital records are complete, and complete follow-up was facilitated by the use of unique national identification numbers for all subjects. The possibility of bias in the selection of data on parity is also unlikely to be a concern.

Taiwan is a small island with a highly developed communications and transportation infrastructure. Furthermore, because of affordable universal health care, it is not unreasonable to believe that virtually all lung cancer patients had access to medical care. In this study, to assess the association between parity and lung cancer we analyzed mortality data rather than data on inpatient admissions. Disease-specific mortality is a function of disease incidence and mortality. Because of the high mortality of lung cancer (it has been reported to have the worst 5-year survival rate of all malignant neoplasms),^2^ lung cancer deaths can be used as a reasonable proxy for lung cancer incidence.

Some studies found that hormone replacement therapy (HRT) may reduce the risk of lung cancer in a population with higher HRT use.^27^^,^^38^^–^^40^ We were unable to adjust for this potential confounder in the current study due to the lack of available data on HRT use. However, since HRT is less frequent in Taiwan than in Western countries,^41^^,^^42^ bias resulting from this factor would be small or absent. Furthermore, if the association between HRT use and lung cancer risk is not as strong as the association between parity and lung cancer risk, adjustment for this variable would not qualitatively change the conclusion.

Cigarette smoking is the most important risk factor for lung cancer.^2^ Unfortunately, there was no information on individual smoking habits and thus it could not be adjusted for in the analysis. However, there is no reason to believe that there would be any correlation between smoking and parity. Furthermore, as mentioned earlier, smoking prevalence among Taiwanese women is very low. Therefore, we believe that not controlling for this variable would have had only a modest, if any, effect on the results. Nevertheless, the problem of possible confounding from smoking should be evaluated. Smoking habits account for at least part of socioeconomic differentials, as rates of smoking are considerably higher among those with lower educational levels. In this study, years of schooling was used as a proxy for socioeconomic status and was included as a control variable in the multivariate analysis. We therefore may have partially indirectly adjusted for the confounding effect of smoking.

There are other risk factors, not included in this study, that could have a role in the pathogenesis of lung cancer, such as passive smoking^43^ and radon.^44^ However, a case-control study in Taiwan reported that exposure to environmental tobacco smoke was not a significant risk factor for lung cancer among nonsmoking women.^45^ To our knowledge, radon is of no relevance in Taiwan. Moreover, there is no reason to believe that there would be any correlation between these risk factors and parity. We postulate that the effect of not controlling for these variables would be modest or nonexistent.

Some potential limitations of this study need to be noted. First, our computerized death-certificate database does not specify the histologic subtype of lung cancer. We therefore could not estimate lung cancer risk in relation to specific histologic subtypes. Second, socioeconomic status is inversely associated with lung cancer risk. We did not have information on the socioeconomic status (eg, income and housing type) of our study subjects, and we could not directly adjust for it in the analysis. However, years of schooling was used as a proxy for socioeconomic status, and this allowed at least partial adjustment for the confounding effect of socioeconomic status. Third, a study of the accuracy of cause-of-death coding in Taiwan found that agreement between reviewers and original coders was high for lung cancer (kappa = 0.98).^46^ Data on the accuracy of lung cancer diagnosis are not available in Taiwan, and misclassification is possible. However, such misclassification is likely to have been nondifferential (ie, unlikely to be related to parity) and therefore would tend to underestimate rather overestimate the true association. Fourth, Taiwan’s vital records and birth registration system cover only live births and exclude stillbirths and abortions. We were therefore unable to examine the possible role of gravidity on lung cancer risk. Fifth, by design, our study focused solely on mortality among parous women. We were unable to examine the possible role of nulliparity on lung cancer incidence. The generalizability of our primary findings to a larger population of all premenopausal women is thus limited. Finally, menopausal status may have been misclassified by using age 50 as the cut-off point. Such misclassification, however, is probably nondifferential and would, if anything, lead to underestimation of our study results.

In summary, premenopausal women of higher parity tended to have an increased risk of lung cancer, although the association was not statistically significant. Despite substantial experimental evidence for the role of hormonal factors in the etiology of lung cancer, the currently available epidemiologic evidence is inconsistent. More work will be needed to clarify the role of parity in lung cancer, and our findings warrant further study.
